# The I148M PNPLA3 Variant Forces Progressive Portal MASLD by Spatially Perturbing Metabolic Pathways Across Liver Zones

**DOI:** 10.3390/ijms27031601

**Published:** 2026-02-06

**Authors:** Erika Paolini, Marica Meroni, Miriam Longo, Sara Badiali, Marco Maggioni, Anna Ludovica Fracanzani, Paola Dongiovanni

**Affiliations:** 1 Medicine and Metabolic Diseases, Fondazione IRCCS Ca’ Granda Ospedale Maggiore Policlinico, 20122 Milan, Italy; erika.paolini@policlinico.mi.it (E.P.);; 2 Department of Surgery, Fondazione IRCCS Ca’ Granda Ospedale Maggiore Policlinico, 20122 Milan, Italy; 3 Department of Pathology, Fondazione IRCCS Ca’ Granda Ospedale Maggiore Policlinico, 20122 Milan, Italy; 4 Department of Pathophysiology and Transplantation, Università degli Studi di Milano, 20122 Milan, Italy

**Keywords:** MASLD, I148M *PNPLA3*, spatial transcriptomic, liver zonation, non-invasive prognosis

## Abstract

Genetics strongly impacts the course of metabolic dysfunction-associated steatotic liver disease (MASLD), with the I148M *Patatin like phospholipase domain containing 3* (*PNPLA3*) variant representing the main modifier. Fat accumulation in the hepatic lobule, strongly enhanced by this SNP, may be influenced by the liver’s zonation. Therefore, we applied spatial transcriptomics to investigate the metabolic processes across portal (PZ)-central (CZ) zones in I148M *PNPLA3* carriers. Visium CytAssist technology was applied to liver biopsies from MASLD patients sharing similar disease severity, who were wild-type (WT) or homozygous for the I148M variant (Discovery cohort, *n* = 4). The distribution of steatosis, inflammation, and fibrosis was assessed in the liver biopsies of MASLD patients, stratified according to the I148M variant (validation cohort, *n* = 100). At the Visium-LOUPE browser, we spatially mapped PZ and CZ hepatocytes (HEPs), revealing higher lipid turnover, glucose signaling, and lower mitochondrial activity in I148M-PZ-HEPs compared to 148M-CZ-HEPs. Thus, the I148M variant could unbalance the physiological hepatic zonation boosting steatosis development in PZ, consequently inducing mitochondrial dysfunction. The unsupervised analysis confirmed the altered metabolic pattern among CZ and PZ in patients carrying the variant. Interestingly, *PNPLA3* expression was higher in I148M-PZ, which also showed an enrichment of non-parenchymal cells, thus possibly explaining the more severe injury in this area. Finally, in the validation cohort, we observed a pronounced PZ distribution of steatosis, inflammation, and fibrosis in I148M *PNPLA3* subjects compared to WT, confirming the spatial data. The I148M variant contributes to the metabolic switching across different hepatic zones and represents a new clinical perspective by defining a specific histological pattern of MASLD.

## 1. Introduction

Metabolic dysfunction-associated liver disease (MASLD) has a worldwide prevalence of 38% and covers a pathogenic spectrum ranging from simple steatosis to steatohepatitis (MASH), cirrhosis, and hepatocarcinoma (HCC) [1,2,3]. MASLD onset is owed to both epidemiological and genetic factors, and it is expected to become the leading cause of end-stage liver disease worldwide [4]. The I148M polymorphism in patatin-like phospholipase domain-containing 3 (*PNPLA3*) gene is considered the strongest genetic predictor of MASLD, claiming a global prevalence of 30–50% [5]. *PNPLA3* is a lipase that is mainly expressed in the liver and adipose tissues, and it is localized on the surface of lipid droplets (LDs), where it catalyzes the hydrolysis of triglycerides. The I148M variation impairs *PNPLA3* enzymatic activity, resulting in the accumulation of the mutated protein on the surface of LD through the ubiquitylation eluding [6,7].

*PNPLA3* expression is mediated by Sterol Regulatory Element Binding Protein 1c (SREBP1c)/ Liver X Receptor (LXR) and Carbohydrate-responsive-element-binding protein (ChREBP) transcription factors, which regulate lipid and glucose metabolism, respectively, suggesting that its activity is subject to nutritional control [4,8]. Moreover, I148M *PNPLA3* carriers display alterations in mitochondrial (mt) functions encompassing de novo lipogenesis, ketogenesis, and β-oxidation, which, in turn, increase the redox state [9]. Consistently, we demonstrated that the overexpression of I148M mutated protein in hepatoma cells led to fat accumulation, impaired mt-lifecycle, and respiration, thus resulting in high oxidative stress. Conversely, the restoration of wild-type (WT) *PNPLA3* activity reduced LDs accumulation, rescued mt-function, and improved hepatocellular injuries [10].

The application of spatial omics could strongly empower the understanding of metabolic processes within liver zones and their impact on MASLD progression. Notably, hepatocytes (HEPs) work in repetitive hexagonal lobule structures centered on the branch of the hepatic central vein. The portal triad encompassing hepatic artery, portal vein, and bile duct is located in the hexagonal corners, ensuring that blood flows inward across the radial axis, generating a hormones, nutrients, and oxygen gradient from portal zone (PZ, zone 1) to central one (CZ, zone 3) towards an intermediate region (zone 2) [11,12]. This gradient impacts on HEPs zonation, affecting their transcriptome profiles, metabolic features, susceptibility to damage, and mt-morphology and function [13,14]. To guarantee physiological metabolism, HEPs require intrahepatic crosstalk with non-parenchymal cells (NPCs) within distinct zones [15,16].

Zonal metabolism may be disrupted by fat accumulation occurring in MASLD. Notably, steatosis originates in the PC zone in response to localized distribution of fatty acids synthesis. Similarly, MASH begins in the PC area, due to higher oxidative stress and hepatocellular injury, and subsequently diffuses throughout the entire lobule. Specifically, PC HEPs induce lipogenesis, whereas PP ones down-regulate β-oxidation and foster the expression of PC genes, including those involved in de novo synthesis of lipids. In this process named periportal-to-pericentral reprogramming zone 2 HEPs come to acquire the features of zone 1, thus favoring the progression towards advanced disease stages and involves the interaction between HEPs and NPCs [17,18].

Prompted by these novel insights and in the attempt to investigate the impact of the I148M *PNPLA3* mutation on liver zonation, we conducted spatial transcriptomics in liver biopsies of MASLD patients featuring similar disease severity, which were WT or homozygous for the variant, mainly focusing on the metabolic changes within PZ and CZ.

## 2. Results

### 2.1. The I148M Polymorphism Spatially Rearranges the Hepatic Metabolism

**Figure 1:**
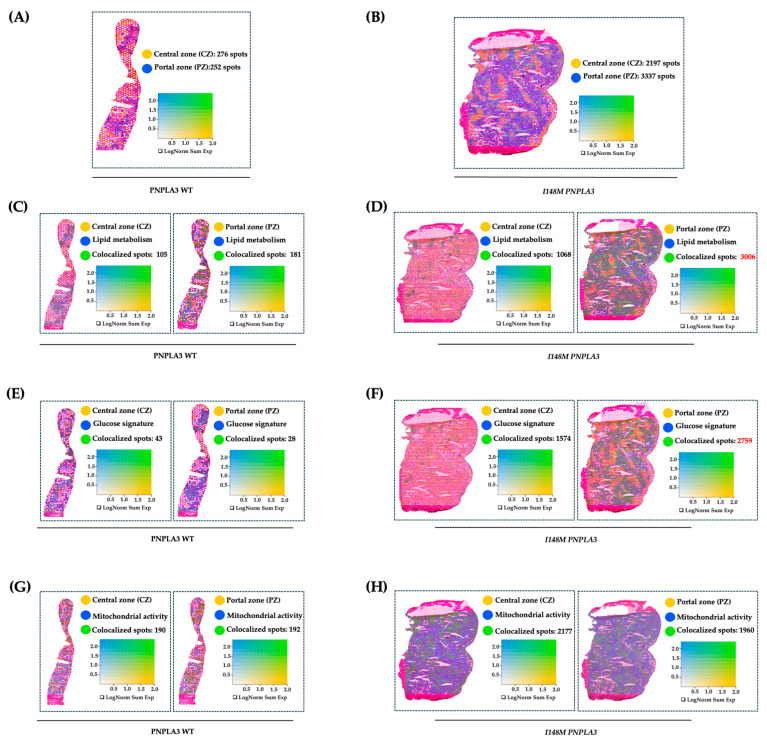
The I148M variant impairs metabolic zonation: (**A**,**B**) Spatial feature plots show the expression of CYP3A4 (CZ, in yellow) and SDS (PZ, in blue) genes across the liver sections of WT (**A**) and I148M *PNPLA3* (**B**). The colocalization map highlights the spatial co-expression of the UMI counts of marker genes of zonation (indicated in Table S2) as logarithmic normalization (LogNorm), thus discriminating CZ and PZ (LOUPE browser). The colocalization is evidenced by green spots. (**C**–**H**) Spatial feature plots show the expression of genes (indicated in Table S2) involved in lipid metabolism, glucose metabolism, and mt-activity (in blue). These pathways were zonated by co-expressing the UMI counts of CZ or PZ signatures (in yellow). The colocalization is evidenced by green spots. Features (LOUPE): avg_log2FC > 0.5, *p*_val_adj < 0.05. LogNorm displays the UMI counts normalized to the total number of UMI counts per spot to compare the summed expression levels of all features in the list (Sum) between spots.

To further investigate how the variant in *PNPLA3* affects metabolic zonation, we conducted an experiment of spatial transcriptomics in liver biopsies of WT and I148M homozygous patients with similar disease severity. We exploited Loupe Browser to zone HEPs and their metabolic signatures in specimen areas. We defined CZ and PZ as two non-overlapping areas within WT and I148M *PNPLA3* (Figure 1A,B) samples by using established markers of zonation [19]. Next, we plotted panels of genes belonging to lipid synthesis, glucose metabolism, and cellular damage possibly mediated by mt-dysfunction in blue (Table S2), which were spatially merged with yellow CZ or PZ signatures, resulting in colocalized green spots (Figure 1C–H). Although both WT and I148M patients featured the same grade of steatosis, we noted a prominent distribution of lipid metabolism-spots in CZ and PZ of WT individuals (Figure 1C). Conversely, lipid metabolism was enhanced, especially in I148M-PZ, revealing a potential role of the pathogenic variant in inducing fat accumulation in this area (Figure 1D). Accordingly, genes involved in glucose metabolism were physiologically expressed in WT-CZ declining towards PZ, while they were strongly upregulated in I148M-PZ (Figure 1E,F). Concerning cellular damage, CZ and PZ of WT patients counted a similar number of spots related to mt-activity, whereas they declined significantly in I148M-PZ compared to I148M-CZ (Figure 1G,H). To further corroborate the mt-dysfunction observed in I148 patients, we observed that the hepatic oxygen consumption rate (OCR) negatively correlated with the presence of portal disease in I148M individuals (ß = −2565.26; 95%CI: −522.13–−4608.37; *p* = 0.02; Table S4).

Overall, I148M polymorphism spatially rearranged hepatic PZ metabolism by exacerbating lipid turnover, glucose signaling, and inhibiting mt-activity and respiration.

### 2.2. The Unsupervised Analysis Confirmed an Altered Metabolic Pattern in Central and Portal Zones in I148M PNPLA3 Carriers

**Figure 2:**
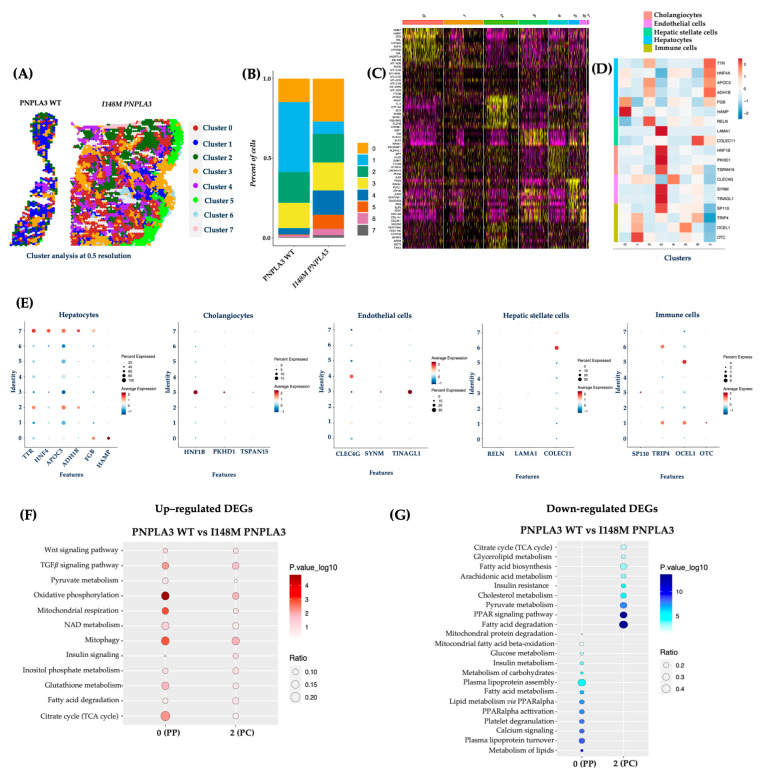
The unsupervised cluster analysis confirmed an altered I148M metabolic pattern: (**A**) The unsupervised analysis at 0.5 resolution allowed the identification of seven clusters, which are represented by the spatial feature plots. (**B**) The Bar Plot of the integrated samples showed an enrichment of clusters 0, 4, 5, 6, and 7 in I148M *PNPLA3* samples compared to WT *PNPLA3*. (**C**) The heatmap exhibits the top marker genes of seven integrated clusters after setting *p* < 0.05 and log2 fold change > 0.5 as thresholds. (**D**) The heatmap displays the expression levels of selected marker genes (*y*-axis), which define cell types across the identified clusters (*x*-axis) by unsupervised analysis. Gene expression values are scaled (z-score) across rows. High and low expression are in red and blue, respectively. (**E**) The dot plot showed the expression of specific genes for HEPs and NPCs in the different clusters (0–7). In the y-axis, the cluster identity is shown, whereas the term Features in the x-axis indicates genes. Dot size represents the percentage of spatial spots within a cluster that expresses a gene, whereas dot color intensity indicates the average expression level of that gene across all spatial spots in the cluster. (**F**,**G**) Dot Plot representing the KEGG pathway-enrichment analysis performed by exploiting DEGs (avg_log2FC > 0.5, *p*_val_adj < 0.05) among WT *PNPLA3* up-regulated DEGs (red) vs. I148M *PNPLA3* down-regulated DEGs (blue) of cl-0-PP and cl-2-PC. The dot size indicates the k/*n* ratio (Ratio), where k is the number of genes participating in the KEGG pathway and *n* is the number of genes annotated as participants of any KEGG pathway. The dot color indicates the enrichment test FDR (Fisher’s exact test).

To validate the Loupe Browser results, we analyzed the data obtained from the integrated dataset, and the top 15 significant principal components were subjected to cluster (cl) analysis. This unsupervised analysis allowed the identification of 7 cl (from 0 to 7) and the spatial feature plots visualized them in both WT and I148M liver biopsies (Figure 2A). Cluster distribution differs across the two genotypes, showing an enrichment of cl 0, 4, 5, 6, and 7 in the *PNPLA3* samples compared to the WT ones (Figure 2B). Since each spot includes more cell types featuring similar transcription profiles, the clusters’ identity appeared heterogenous (Figure 2C). Therefore, the top marker genes derived from the integrated process (Figure 2C) and the ascertained cell-type signatures (Table S2) were combined to annotate cell populations. Furthermore, both gene lists were exploited to conduct a GO enrichment, and clusters were labeled as follows: cl0, HEPs; cl1, immune cells; cl2, HEPs; cl3, cholangiocytes, endothelial cells (ENDOs); cl4, ENDOs; cl5, immune cells; cl6, HSCs and immune cells; cl7, HEPs (Figure 2C–E).

Concerning the link between the *PNPLA3* variant and unbalanced metabolic zonation, we exploited the integrated top marker gene list to firstly define cl0 as PZ and cl2 as CZ, while cl7 included HEPs without differences between PZ and CZ in the expression of marker genes (Cl0, PZ: *NNMT*, *HAMP*, *SDS*, *HAL*, *CYP3A5*, *ASPG*, *CYP2B6*, *ASL*, *ANGPTL4*; Cl2, CZ: *APOA2*, *AMBP*, *CLU*, *CYP1A2*, *SCD*, *AHSG*, *EPHX1*, *ALDH2*, *CYP2E1*; Figure 2C).

DEGs among clusters 0 and 2 were employed to perform pathway-enrichment analysis by using the KEGG database. In keeping with the canonical zonation, WT *PNPLA3* biopsies exhibited an enrichment of pathways related to pyruvate metabolism, oxidative phosphorylation, mt-respiration, NAD metabolism, mitophagy, and TCA cycle in PZ-cl0, which gradually declined in CZ-cl2 (Figure 2F). Conversely, DEGs belonging to I148M-PZ-cl0 were predominantly enriched in genes involved in glucose, and much more in lipid metabolism, showing a complete lack of mt-activity. Interestingly, I148M-CZ-cl2 displayed a specular metabolic signature (Figure 2G). In summary, these data reinforced the contribution of the *PNPLA3* variant in impairing the metabolic zonation, which in turn could prompt more severe liver damage in specific areas.

### 2.3. I148M Carriers Displayed Higher PNPLA3 Expression in Portal Zone

**Figure 3:**
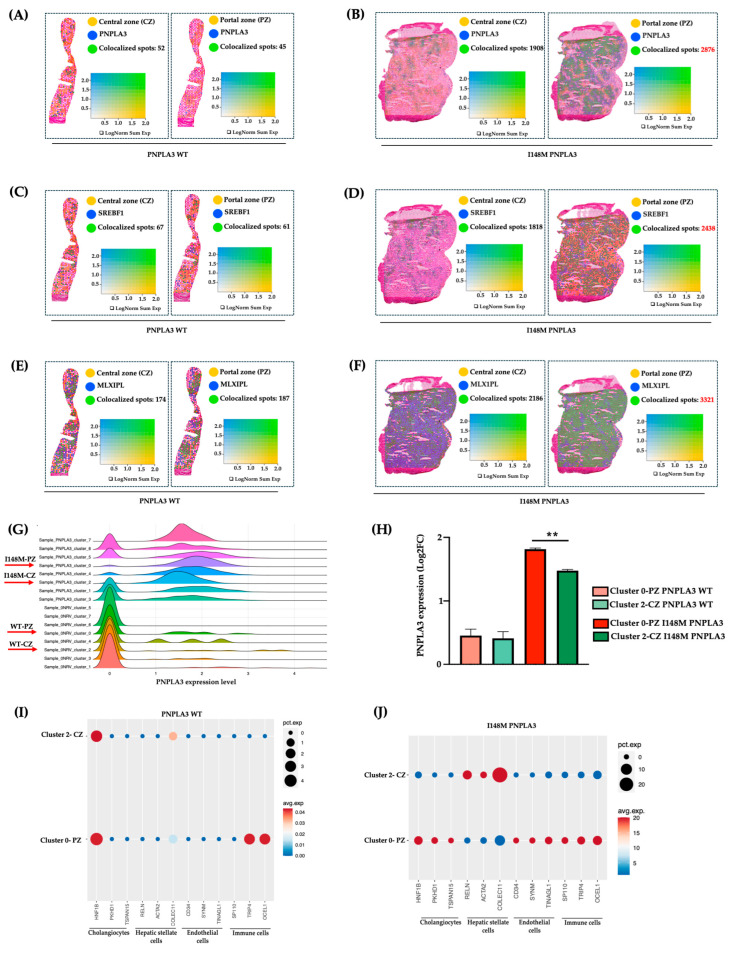
*PNPLA3* expression increased in I148M-PZ: (**A**–**F**) Spatial feature plots show the expression of *PNPLA3/SREBF1/MLXIPL* genes across the liver sections of WT (**A**,**C**,**E**) and I148M *PNPLA3* (**B**,**D**,**F**). The colocalization map highlights the spatial co-expression of the UMI counts of *PNPLA3*, *SREBF1*, and *MLXIPL* (in blue) with markers of zonation (in yellow, indicated in Table S2) as logarithmic normalization (LogNorm) of WT (**A**,**C**,**E**) and I148M *PNPLA3* (**B**,**D**,**F**). (avg_log2FC > 0.5, *p*_val_adj < 0.05). LogNorm displays the UMI counts normalized to the total number of UMI counts per spot to compare the summed expression levels of all features in the list (Sum) between spots. The colocalization is evidenced by green spots. (**G**) The ridge plot showed the distribution of *PNPLA3* expression (avg_log2FC) among each integrated clusters WT *PNPLA3* and I148M *PNPLA3* samples. Each curve represents a cluster, with the density reflecting the proportion of spots expressing the *PNPLA3* gene in WT and I148M *PNPLA3* samples. (**H**) The bar graph of *PNPLA3* expression in cl-0-PZ and *cl-2-CZ* of WT *PNPLA3* (salmon and light green) and I148M *PNPLA3* (red and dark green) (avg_log2FC + SE, adjusted, ** *p* < 0.01 at Tukey’s honestly significant difference (HSD)). (**I**,**J**) The Dot Plot shows the expression of classical cell-type marker genes for HEPs and NPCs (*x*-axis) across cl0 and cl2 (*y*-axis) of the WT *PNPLA3* and I148M *PNPLA3* samples. Dot size represents the proportion of spatial spots within that cluster expressing the gene (pct.exp), whereas dot color indicates the average expression level among the expressing spots (avg.exp).

To investigate whether the altered zonation was due to a different *PNPLA3* gene expression across the hepatic lobule, we evaluated its mRNA levels in PZ-cl0 and CZ-cl2 of WT and I148M patients. We observed that *PNPLA3,* alongside its transcriptional factors *SREBF1* and *MLXIPL* (*CHREBP*), which regulate lipid and glucose metabolism, respectively, were equally expressed between WT-PZ-cl0 and WT-CZ-cl2 (Figure 3A–C,G–H), Conversely, they increased in I148M-PZ-cl0 compared to I148M-CZ-cl2 (Figure 3B–D,G–H). These findings suggest that the spatial re-arrangement of metabolic functions associated with the presence of the *PNPLA3* variant may be due to its increased expression mainly in PZ.

Next, to assess whether the different expression of the *PNPLA3* gene in PZ and CZ may impact progressive damage, we investigated in clusters 0 and 2 of WT and *PNPLA3* samples, the distribution of NPCs, involved in inflammation and fibrosis. Indeed, these two clusters of HEPs also included a fraction of NPCs, sharing the same zonation. We revealed that NPCs were equally distributed in WT-CZ-cl2 and WT-PZ-cl0, except for the immune population, which was mainly located in PZ-cl0, possibly deriving from the portal vein (Figure 3I). Conversely, NPCs mainly including cholangiocytes, ENDOs, and immune cells were enriched in I148M-PZ-cl0 compared to I148M-CZ-cl2 (Figure 3J). Notably, HSCs were predominant in I148M-CZ-cl2, mainly localized at the mid-lobular and pericentral regions, specifically in the perisinusoidal area between ENDOs and hepatocytes, and their different spatial zonation correlate with functional differences. However, we cannot rule out the presence of activated myofibroblasts in the PZ, which were not identified by the analysis.

These data were confirmed by Loupe browser, through which we simultaneously plotted the signatures related to HEPs and NPCs, proving colocalized spots, especially in I148M-PZ-cl0 (Figure S2). Overall, we could speculate that NPCs were mainly recruited in I148M-PZ-cl0, compared to WT, potentially contributing to disease severity in this specific area.

### 2.4. The I148M Variant Contributes to the Severity of Periportal Damage

**Figure 4:**
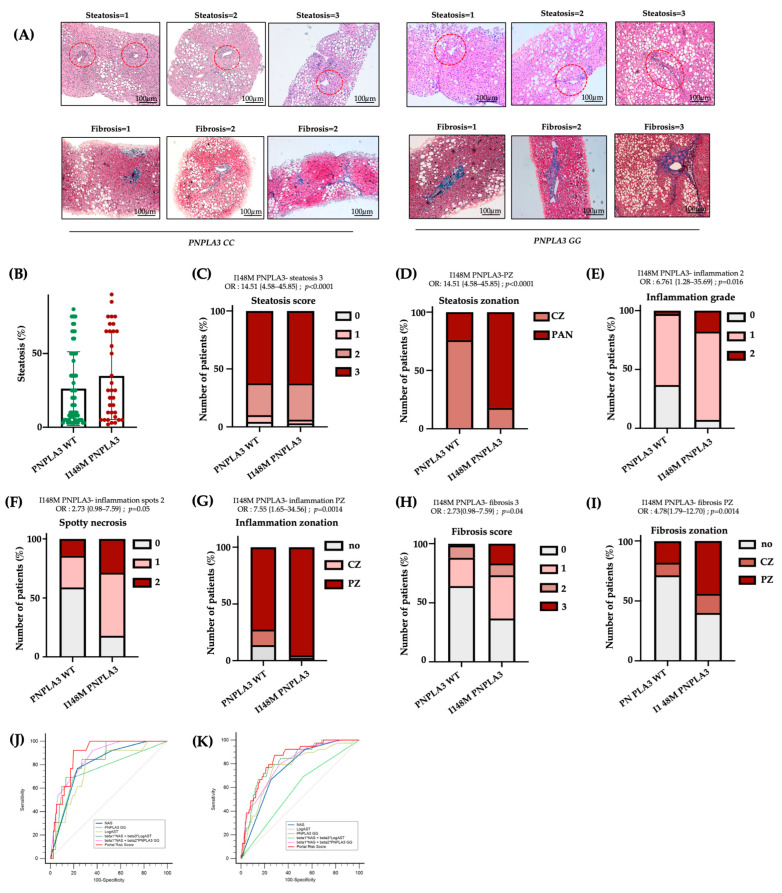
The I148M variant defines periportal injury: (**A**) Hematoxylin-Eosin and Masson’s Trichrome staining were performed in *n* = 100 hepatic tissues. Representative images of steatosis, inflammatory foci and fibrotic septa distribution within hepatic zones. Red circle underlined PZ (Original magnification 100×, scale bar 100 µm). (**B**) Percentage of steatosis in *PNPLA3* -WT (*n* = 66) and *PNPLA3* homozygous (*n* = 34) patients. (**C**–**I**) Univariate analysis (not adjusted) of the *PNPLA3* _I148M variant with the presence of steatosis degree = 3 (**C**), OR = 14.51 {4.58–45.85}; *p* < 0.0001, PZ-steatosis (**D**), OR = 14.51 {4.58–45.85}; *p* < 0.0001, inflammation grade = 2 (**E**), OR= 6.761 {1.28–35.69}; *p* = 0.016, spotty necrosis (**F**), OR = 2.73 {0.98–7.59}; *p* = 0.05, PZ inflammation (**G**), OR = 7.55 {1.65–34.56}; *p* = 0.0014, fibrosis score = 3 (**H**), OR = 2.73 {0.98–7.59}; *p* = 0.04 and PZ-fibrosis (**I**), OR = 4.78 {1.79–12.70}; *p* = 0.0014. (**J,K**) Receiver operating characteristic (ROC) curves (sensitivity vs. 100–specificity) comparing the diagnostic performance of different risk models: NAS, *PNPLA3* GG, LogAST, combined models (beta1*NAS + beta3*LogAST and beta1*NAS + beta2*PNPLA3 GG), and the Portal Risk Score in the Validation (**J**) and Independent Retrospective (**K**) cohorts. The diagonal line represents the performance of a random classifier.

To confirm the presence of more severe damage in I148M-PZ, we scored steatosis, inflammatory foci, and fibrotic septa distribution in *n* = 100 biopsies from MASLD patients (Validation cohort, Table S1). By comparing I148M *PNPLA3* and WT patients, we found that steatosis displayed a more widespread distribution in I148M homozygous individuals compared to WT (at bivariate analysis *p* < 0.0001; Figure 4A–D). In nominal logistic regression analysis adjusted for sex, age, BMI, diabetes and disease severity, the I148M polymorphism was significantly associated with panlobular steatosis (OR = 16.23; 95%CI: 4.12–63.89; *p* < 0.0001; Table S3). Conversely, the grade of inflammation, spotty necrosis, and fibrosis were higher in I148M *PNPLA3* subjects compared to WT, exhibiting an increased periportal distribution (at bivariate analysis *p* < 0.05 for all comparisons, Figure 4A–I). In nominal logistic analysis adjusted as above, the I148M polymorphism was independently correlated with periportal inflammation and fibrosis (periportal inflammation: OR = 10.76; 95%CI: 1.78–64.84; *p* = 0.0028; periportal fibrosis: OR = 4.33; 95%CI: 1.23–15.1; *p* = 0.0182; Table S3). Furthermore, immunohistochemistry evaluation showed that the *PNPLA3* protein expression was higher in hepatic tissues of I148M patients compared to WT ones, not only in HEPs but also in NPCs localized in PZ (Figure S3A).

Consistently, in a subgroup of patients belonging to the Validation cohort (*n* = 45), for whom bulk transcriptomic data were available, we assessed the hepatic expression of *PNPLA3*. We revealed that it positively correlated with periportal fibrosis in I148M patients compared to WT (ß = 0.59; 95%CI: 0.20–0.98; *p* = 0.004; Table S5). These data support the notion that patients with the presence of portal fibrosis showed the highest expression of mutated *PNPLA3*, which may contribute to zonated progressive injuries. Accordingly, transcriptomic analysis revealed that *SREBF1* and *MLXIPL* (*CHREBP*), alongside genes involved in lipid and glucose metabolism, were significantly upregulated in I148M patients featuring periportal fibrosis (Figure S3B), further confirming the spatial results.

Finally, in the attempt to create a new score which considers not only NAS but also the zonal disease distribution, genetic background and clinical features, we built a new Portal Risk Score and tested its performance in the Validation cohort (*n* = 100). Since our findings demonstrated that GG carriers displayed predominant portal pattern of disease, even in the presence of low NAS values, we assessed whether the Portal Risk Score may predict the presence of fibrosis >2 more efficiently than NAS in the Validation cohort stratified according to the presence of NAS < 4. The new score was constructed by weighting NAS together with *PNPLA3* genotype and serum AST levels within a multivariable logistic regression model using portal disease as reference. In patients with NAS < 4, our score displayed a high discriminative performance for advanced fibrosis compared to NAS alone with an AUC of 0.89 vs. 0.80 for NAS. At the optimal cut-off (Youden index), the Portal Risk Score achieved higher sensitivity than NAS (93% vs. 77%), while specificity was comparable (23% vs. 20%) (Figure 4J, Table S6). The overall diagnostic performance was higher (Youden index 0.73 vs. 0.54), although the comparison between the two ROC curves did not reach statistical significance (*p* = 0.11 Portal Risk Score vs. NAS). This discrepancy may be explained by the limited sample size of the Validation cohort (*n* = 100). 

Therefore, we next decided to validate the efficacy of Portal Risk Score in the large independent historical cohort of 1466 patients (Independent retrospective cohort). In these patients, we confirmed that our score showed a superior performance in discriminating advanced fibrosis in patients with NAS < 4 (AUC = 0.85 vs. 0.77 for NAS; sensitivity = 87% vs. 71% for NAS; specificity= 23% vs. 25% for NAS; Youden index = 0.60 vs. 0.46 for NAS; *p* = 0.0089 Portal Risk Score vs. NAS; Figure 4K, Table S7). These findings suggest that at lower level of histological liver damage, NAS alone may underestimate the risk of advanced fibrosis, whereas the new Portal Risk Score displays enhanced sensitivity and captures additional susceptibility conveyed by the *PNPLA3* genetic background.

## 3. Discussion

MASLD exhibits a specific hepatic zonation. Indeed, in the early stages, steatosis and inflammation are localized pericentrally, whereas in the late stages, they are more diffuse across the parenchyma. Differently from fibrosis, whose stage and localization are defined according to Kleiner et al. [20], the NAS score is unable to zone steatosis, necroinflammation, and ballooning. Nevertheless, the increased portal distribution of the disease is significantly associated with worse outcomes, and risk of liver transplantation. Therefore, it is necessary to identify novel scoring strategies that consider the positional cellular/metabolic changes occurring during disease progression. In this context, data obtained from novel spatial approaches could aid the standard systems in MASLD diagnosis and prognosis.

In physiological conditions, HEPs’ functions guarantee a gradient of hormones, nutrients and oxygen from PZ toward CZ, thus refining their transcriptome profiles and metabolic activities. In this regard, PZ-HEPs are responsible for glucose metabolism, gluconeogenesis, fatty acid oxidation, and cholesterol synthesis, while CZ-HEPs are involved in glucose uptake, glycolysis, lipogenesis, and ketogenesis [21]. Furthermore, to assure metabolic heterogeneity in different zones, HEPs interact with ENDOs, Kupffer cells, HSCs, cholangiocytes, and immune cells. Therefore, zonation is essential for liver physiology, and its disruption may be closely associated with MASLD progression.

The I148M *PNPLA3* mutation is the strongest genetic determinant of MASLD, and it acts by impairing the hydrolysis of triglycerides [4,10]. MASLD patients carrying the G allele show a specific histological pattern, depicted by macro/microvesicular steatosis, portal inflammation, proliferation of hepatic progenitor cells, ductular reaction, and myofibroblast activation, thus sustaining fibers deposition in the portal zone [22]. Although the *PNPLA3* variant has been associated with worse hepatic damage, few studies have investigated the potential mechanisms through which this mutation affects hepatic zonation.

In this study, we performed spatial transcriptomics to assess metabolic differences among CZ and PZ of WT and I148M patients, both with similar disease severity. Notably, the I148M *PNPLA3* variant promotes triglyceride accumulation, which can initially affect CZ-HEPs. This could induce further dynamic adaptation in fatty acid metabolism, resulting in the early spreading of CZ-steatosis within the lobule [23]. Carpino and colleagues demonstrated that patients carrying the I148M variant had higher steatosis and inflammation in PZ-HEPs compared to WT individuals [22]. Consistently, our LOUPE data revealed that WT patients exhibited an enriched lipid metabolism in both CZ and PZ, which was exacerbated in I148M patients, especially in PZ, revealing its zone-specific pathogenic activity. Similarly, we detected higher glucose metabolism in PZ-HEPs of I148M patients compared to WT, thus corroborating the impact of this variation on metabolic pathways even more in PZ. Accordingly, SREBP and ChREBP expression, which, in turn, regulates that of *PNPLA3*, was increased in PZ [24]. Similarly, bulk transcriptomics confirmed spatial results by highlighting an increased expression of *PNPLA3*, of its transcription factors, and of genes implicated in lipid and glucose metabolism in I148M patients characterized by periportal disease.

I148M *PNPLA3* overexpression in Huh-7 cells was correlated with high levels of lactate and glutamyl-amino acids, which are hallmarks of metabolic switching and mt-dysfunction, respectively. Recently, we demonstrated that the I148M overexpression in HepG2 cells enhanced fat accumulation and reduced OXPHOS rate and ATP production. This boosted oxidative stress as well as the release of lactate and ccf-mtDNA, overall contributing to disease severity [10]. Consistently, spatial data revealed that WT patients exhibited a physiological metabolism within hepatic zones characterized by high mt-activity in the PZ-HEPs, which gradually decreased in the CZ-HEPs. Conversely, I148M carriers had a subversion in oxidative functions with a predominance of mt-activity in CZ-HEPs. Prompted by these observations, we investigated the association between the mt-respiration and portal disease, showing that OCR was particularly reduced in I148M patients with periportal fibrosis. Overall, the switching in the bioenergetic metabolism could explain the injury detected in the PZ.

Previous studies have reported that the different mt-activity might be paralleled by structural changes of the organelles. Indeed, the latter may be shaped by nutrient, hormone, and oxygen availability, thus revealing a selective zonation in the liver. Specifically, central organelles appear tubular-shaped since they are involved in enzymatic activity such as lipid and carbohydrate metabolism, citrate synthase activity, and TCA cycle. Conversely, portal mitochondria exhibit spherical morphology related to amino acid metabolism, OXPHOS activity, ATP production, mitophagy, and oxygen gradient [15,25]. We recently observed that mt-morphology is compromised during severe MASLD, and more so in genetically predisposed individuals [15,26]. It is conceivable that mitochondria located in PZ-HEPs cannot counteract the fat load with the consequent assembly of failed organelles with low respiration capacity. As a compensatory mechanism, mitochondria belonging to CZ-HEPs try to stimulate mitobiogenesis to discard fat accumulation in PZ. To explain the mechanisms through which the *PNPLA3* variant impacts on liver zonation, we investigated its gene expression across PZ and CZ, and we observed higher *PNPLA3* mRNA levels in PZ of I148M patients according to increased *SREBF1* and *MLXIPL* (*CHREBP*) expression. It could be hypothesized that the accumulation of the mutated protein in PZ may trigger fat buildup in this area. In turn, it might alter retinol release from HSCs, thus precipitating fibrogenesis. Our observations are in line with recent data from Watson et al., who spatially defined hepatocytes into zones 1, 2, and 3 across the lobule of healthy livers and compared their pattern of expression to fibrotic ones. In the latter, they identified portal (zones 1 and 2) and central (zone 3) hepatocytes, thus suggesting that, during chronic liver injury, the zonation is subverted. Moreover, in fibrotic hepatocytes (Hep 1) the authors observed high *PNPLA3* expression as a consequence of advanced liver injury [12].

Literature evidence demonstrated that patients carrying the I148M *PNPLA3* are characterized by the activation of the hepatic stem cells/progenitor cell niche [22,27]. The latter is composed of macrophages, HSCs, and well-defined extracellular matrix compounds that overall contribute to portal fibrosis. We detected more cholangiocytes, ENDOs, and immune cells in I148M patients compared to WT ones. Indeed, NPCs co-zoned with PZ-HEPs in I148M patients unless for HSCs, which were predominantly located in I148M-CZ according to their physiological hepatic distribution in the Space of Disse [28,29]. It has been reported that I148M variant directly enhances pro-inflammatory and profibrogenic gene expression in HSCs, disrupts lipid metabolism, and causes mitochondrial dysfunction and ROS generation, linking metabolic stress to fibrogenesis [30,31,32]. The accumulation of toxic metabolites, oxidative stress, and altered nutrient sensing in PZ, may in turn activate resident immune cells and amplify pro-inflammatory cytokine release [17]. In sum, our results underline that the portal pattern of liver damage encompassing subverted metabolism and enrichment of NPCs is a key feature in patients carrying the I148M variant.

Several studies exhibited that PZ inflammation is present in 60–80% of adult patients, suggesting that portal involvement is associated with more advanced disease [33]. It has been observed that portal inflammation and fibrosis in adult MASLD over a 13-year follow-up were associated with increased risk of death or liver transplantation. Conversely, MASLD patients without periportal fibrosis were strongly protected from cirrhosis-related complications. Accordingly, our results showed that among 100 MASLD patients (Validation cohort), the I148M polymorphism was significantly associated with panlobular steatosis, whereas the grade of inflammation, spotty necrosis and fibrosis were higher in I148M *PNPLA3* subjects, compared to WT individuals, exhibiting a more periportal distribution. Moreover, the I148M polymorphism was independently associated with periportal inflammation and fibrosis, thus corroborating the direct role of I148M *PNPLA3* variant in driving the portal damage, as described by Carpino et al. [22]. Moreover, we revealed that I148M *PNPLA3* patients had increased hepatic mRNA and protein levels of *PNPLA3* and the latter correlated with the presence of periportal disease. Since both the I148M *PNPLA3* variant and periportal disease represent prognostic risk factors for the development of fibrosis/cirrhosis and considering the increased expression of *PNPLA3* in PZ, we tried to build a new score (Portal Risk Score) which integrates NAS, zonal disease distribution, genetic background and clinical features. Next, we tested its clinical utility in predicting advanced fibrosis in patients with NAS < 4. We demonstrated that the Portal Risk Score may foresee the presence of fibrosis >2 more efficiently than NAS in both Validation and Independent Retrospective cohorts. 

The main limitation of this study relies on the modest number of samples used for spatial transcriptomics; nevertheless, this is the first study which combines spatial transcriptomics with genetic stratification (I148M *PNPLA3*) in human MASLD. This small sample size reflects the stringent selection criteria, the high cost and technical complexity of spatial transcriptomics. To improve the exploratory nature of the study, we validated our findings in large independent cohorts thus strengthening the biological relevance and robustness of our spatial results. However, further histological and spatial validations in a large cohort will be required to fully confirm and extend our observations. Despite this limitation, overall, these findings support a model in which the *PNPLA3* variant may amplify portal disease offering novel insights into how genetic variants may alter hepatic zonation.

## 4. Materials and Methods

### 4.1. Patients

We performed spatial transcriptomics in hepatic frozen samples obtained from MASLD patients (*n* = 2 WT, *n* = 2 I148M homozygotes; Discovery cohort; Table S1). We evaluated Hematoxylin-Eosin and Masson’s Trichrome staining (Sigma-Aldrich, St. Louis, MO, USA) in FFPE hepatic tissues of *n* = 100 MASLD patients (validation cohort; Table S1) to score steatosis, inflammatory foci, and fibrotic septa distribution.

Informed written consent was obtained from each patient, and the study protocol was approved by the Ethical Committee of the Fondazione IRCCS Ca’ Granda, Milan.

### 4.2. Spatial Transcriptomics—Visium CytAssist

Tissue preparation, fixation, destaining, probe hybridization, library preparation, and sequencing are extensively described in the Supplemental Material and Methods section [10,20,21,26,34].

### 4.3. Loupe Browser Analysis

First, we have manually selected spots that are covered by hepatic biopsies and employed the filtering and reanalysis workflow in Loupe Browser v8.1.2 (Pleasanton, CA, USA) to conduct quality control based on Unique Molecular Identifiers (UMI) counts, number of detected genes, and percentage (%) of mt-genes. Thus, we set thresholds of at least 50,000 reads/spot (UMI per Barcode as Log2), 400 genes/spot (genes per Barcode as Log2), and 20% of mitochondrial UMI to remove outliers featured by low expression counts or doublets. Next, we performed re-clustering analysis using the K-means value, which provides unsupervised clustering. For cell-type annotation, CZ, PZ, and metabolic signatures (Table S2), the UMI counts of selected genes were expressed as logarithmic normalization (LogNorm), which is a quantitative interpretation to compare the expression of multiple genes across cells. The UMI counts were normalized to the total amount of UMI/spots. To spatially map the co-expression of two genes or two signatures, we exploited the co-expression view function, which plotted a separate feature list by using a distinct color scale as LogNorm (Figure S1).

### 4.4. Statistical Analysis

Data are represented as data points ± SD. Statistical analyses were performed using JMP 16.0 Pro (SAS, Cary, NC, USA), R software (v.3.3.2), and GraphPad Inc., v10 (San Diego, CA, USA). One-way analysis of variance (ANOVA) or the chi-square test was applied when appropriate. p-values were corrected for multiplicity by Tukey’s honestly significant difference (HSD) multi-comparison post hoc test, and adjusted *p*-values < 0.05 were considered statistically significant. For spatial transcriptomics, *p*-values were corrected for multiplicity by the Benjamini–Hochberg method, and adjusted *p* < 0.05 were considered statistically significant. For descriptive statistics, continuous variables are shown as the mean and SD or the median and interquartile range for highly skewed biological variables (i.e., ALT). Variables with skewed distributions were logarithmically transformed prior to the analysis. Multinomial generalized, logistic, and ordinal regression models were fitted to examine continuous, binary, and ordinal traits. When specified, confounding factors were included in the model. *p*-values < 0.05 (two-tailed) were considered statistically significant.

## 5. Conclusions

To conclude, our study shows that I148M patients are characterized by high *PNPLA3* expression in the periportal zone, resulting in subverted metabolism and enrichment of NPCs in this area. The *PNPLA3* variant may represent a non-invasive zonal index of portal injury, thus implementing the NAS score, which does not consider the zonal distribution of the hepatic damage or the presence of fibrosis. New scores integrating NAS with portal disease, transaminases, I148M *PNPLA3* mutation may overcome NAS alone in detecting advanced fibrosis even in MASLD patients with mild disease. 

## Data Availability

The original contributions presented in this study are included in the article/Supplementary Material. Further inquiries can be directed to the corresponding author.

## Supplementary Materials

The following supporting information can be downloaded at: https://www.mdpi.com/article/10.3390/ijms27031601/s1.

## Abbreviations

The following abbreviations are used in this manuscript:

MASLD	Metabolic dysfunction-associated liver disease
MASH	Metabolic dysfunction-associated steatohepatitis
HCC	hepatocellular carcinoma
*PNPLA3*	patatin-like phospholipase domain-containing 3
LDs	lipid droplets
ROS	oxidative reactive species
mt-ccf	mitochondrial DNA fragments
HEPs	hepatocytes
PZ	Portal zone
CZ	Central zone
NPCs	non-parenchymal cells
WT	wild-type
T2DM	Type 2 Diabetes Mellitus
H&E	Hematoxylin-Eosin
BMI	body mass index
ALT	alanine aminotransferase
UMI	Unique Molecular Identifiers
HSCs	hepatic stellate cells
GO	Gene Ontology
TCA	citrate cycle
NAS	NAFLD Activity Score
TM6SF2	transmembrane 6 superfamily member 2
MBOAT7-TMC4	Membranebound O-acyltransferase domain containing 7-transmembrane channel-like 4
HPCs	hepatic progenitor cells
HpSC	hepatic stem cells/progenitor cell
