# Supplementary material for: The I148M PNPLA3 Variant Forces Progressive Portal MASLD by Spatially Perturbing Metabolic Pathways Across Liver Zones
Source: Int J Mol Sci. 2026 Feb 6;27(3):1601. doi: 10.3390/ijms27031601 (PMC12897613; doi:10.3390/ijms27031601)
Supplement: Supplementary file 1 [file ijms-27-01601-s001.zip › ijms-4089114-supplementary.pdf]

The I148M PNPLA3 variant forces progressive MASLD by perturbing metabolic pathways across liver zones

Supplementary Methods

Patients

We performed spatial transcriptomics in hepatic frozen samples of MASLD patients (n=2 WT, n=2 I148M homozygotes; Discovery cohort; ***Table S1***). We evaluated Hematoxylin-Eosin and Masson's Trichrome staining in FFPE hepatic tissues of n=100 MASLD patients (Validation cohort; ***Table S1***) to score steatosis, inflammatory foci, and fibrotic septa distribution. MASH was defined by the presence of steatosis, lobular inflammation, and hepatocellular ballooning. The inclusion criteria were the availability of a liver biopsy for suspected MASH, DNA samples, and clinical data. Individuals with increased alcohol intake, viral and autoimmune hepatitis, hereditary haemochromatosis, and alpha1-antitrypsin deficiency or other causes of liver disease were excluded. Informed written consent was obtained from each patient, and the study protocol was approved by the Ethical Committee of the Fondazione IRCCS Ca' Granda, Milan (ID: 4265) and conforms to the ethical guidelines of the 1975 Declaration of Helsinki. All patients were previously genotyped for rs738409 (PNPLA3-I148M) using TaqMan 5′-nuclease assays (Life Technologies, Carlsbad, CA), as previously described [34].

Histological Evaluation

Steatosis was divided into the following 4 categories based on the percentage of affected hepatocytes: 0, 0%–4%; 1, 5%–32%; 2, 33%–65%; and 3, 66%–100%. Disease activity was assessed according to the NAFLD activity score (NAS), with systematic evaluation of hepatocellular ballooning and necroinflammation; fibrosis was staged according to the recommendations of the NAFLD Clinical Research Network [20]. The scoring of liver biopsy specimens was performed by independent pathologists unaware of patient's status and genotype . MASH was diagnosed in the presence of steatosis, lobular necroinflammation, and hepatocellular ballooning. Patients belonging to the Discovery cohort have NAS of 4 encompassing degrees of steatosis=3 and inflammation=1 without the presence of fibrosis and ballooning.

Hematoxylin-Eosin (H&E) staining

Liver specimens were deparaffinized and rehydrated through 100% alcohol, 95% alcohol 70% alcohol and washed with distilled water. Then, hepatic tissues were stained in Hematoxylin for 1 minute, washed with 4-5 changes of tap water and in 1X PBS for 1 minute. The slides were washed again with three changes of distilled water, counterstained in Alcoholic-Eosin for 1 minute, dehydrated through three changes of 95% EtOH and 2 changes of 100% EtOH 1 minute each. Finally, the slides were cleared in three changes of Xylene for 1 minute each and mounted with the coverslip.

Masson's Trichrome staining

Liver specimens were deparaffinized and rehydrated through 100% alcohol, 95% alcohol 70% alcohol and washed with distilled water. Next, hepatic tissues were stained in Biebrich scarlet-acid fuchsin solution for 10-15 minutes, washed in distilled water, and differentiated in phosphomolybdic-phosphotungstic acid solution for 10-15 minutes. The slides were directly transferred to aniline blue solution, stained for 5-10 minutes, rinsed in distilled water, and differentiated in 1% acetic acid solution for 2-5 minutes. Finally, tissues were washed in distilled water, dehydrated quickly through 95% ethyl alcohol, absolute ethyl alcohol, cleared in xylene, and mounted with a resinous mounting medium.

Immunohistochemistry

Immunohistochemistry (IHC) was performed as a semi-quantitative method to assess the expression of PNPLA3 in the livers of 8 patients, wild-type (n=4) or homozygous (n=4) for I148M PNPLA3 genotype. Sections were incubated with PNPLA3 antibody (Abcam, ab188856) 1:75 and subsequently with secondary antibody anti-goat IgG (Santa Cruz, CA, USA – sc2350) 1:350. The IHC reactions were developed with the secondary antibody anti-goat IgG (Santa Cruz, CA, USA – sc2350) 1:350. The IHC reactions were developed with the avidin–biotin peroxidase method by using diaminobenzidine (DAB) as chromogen.

Spatial transcriptomics- Visium CytAssist

Tissue preparation: freezing and embedding

A bath of isopentane and liquid nitrogen was used to freeze either freshly obtained tissue and then embedded in OCT. OCT blocks were cryosectioned in a Microtome CM 1950 LEICA AGPROTECT cryostat (TRIM= 25µm, SECT= 10µm, chamber temperature= -23°C, cutting plane temperature= -14°C) to generate appropriately sized sections in duplicate fixed on standard microscope slides for the Visium

CytAssist Instrument. The tissue was located within the allowable area of microscope slides as follows: 15mm from the short sides and 5mm from the long sides. Thus, tissues were placed within the 6.5mm Capture Area and will be moved to Visium slides by Visium CytAssist machine.

Tissue fixation and Hematoxylin and Eosin (H&E) staining

Slides were removed from -80°C and placed on dry ice and then transfer on the Thermocycler Adaptor with the active surface facing up, and incubate 1 min at 37°C. Then, they were completely immersed in the pre-chilled methanol and incubated for 30 minutes at -20°C. We added 500 µl isopropanol to uniformly cover all tissue sections for 1 minute at room temperature, and then 1ml of Hematoxylin for 7 minutes at room temperature. Different volumes of Milli-Q water were dispensed as follows: 50ml in one 50-ml centrifuge tube/slide, 800ml in Beaker 1, 800ml in Beaker 2 and 800ml in Beaker 3. Slides were immersed 5 times in the water in a centrifuge tube, 15 times in the water in Beaker 1 and 15 times in the water in Beaker 2. We covered all tissue sections with 1ml Bluing Buffer and incubated 2 minutes at room temperature. Then, slides were immersed 5 times in the water in Beaker 2, we aliquoted 1ml Eosin Mix to uniformly cover all tissue sections and incubated 1 minute at room temperature. Finally, slides were immersed 15 times in the water in Beaker 3 and incubated on the Thermocycler Adaptor with the thermal cycler lid open for 5 minutes at 37°C. The slides should be observed at optical microscope to define the area of interest (6.5 X 6.5 mm) compatible with the Visium CytAssist slide in which the tissues will be located.

Destaining

Slides were placed in the Visium CytAssist Tissue Slide Cassette. Weed 150µl of 0.1N HCl along the side of the wells to uniformly cover the tissue sections and then remove it. 150µl of 0.1N HCl were added to the side of the wells to uniformly cover the tissue sections without introducing bubbles. We placed the cassette on the Low-Profile Thermocycler Adapter at 42°C to remove the H&E staining.

Probe hybridization and ligation

Tissue sections were appropriately stained, imaged, and destained. Then, the human whole transcriptome probe panel, consisting of ~3 specific probes for each targeted gene, was pipetted on tissues. Each probe was provided by a poly A tail to be recognized and bound by Visium Gene Expression Bar Coded Spots. The slides were located on the Tissue Slide Cassette with 100µl of Pre-Hybridization Mix for 15 minutes at room temperature. We removed the Pre- Hybridization Mix, added 100µl of Hybridization Mix and placed the slides on the Low-Profile Thermocycler Adapter on the thermal cycler at 50°C overnight. The day after, we washed all tissues with 150µl of Wash Buffer

followed by 5 minutes at 50°C for 3 times. For probe ligation, we treated samples with 150µl of 2X SSC Buffer for 3 minutes at room temperature. After removing the buffer, we placed them on the Low-Profile Thermocycler Adapter on the thermal cycler at 37°C for 1 hour with 60µl of Ligation mix and. Next, we proceeded with the wash steps 3 times as follows: 100µl of Pre-heat Post-Ligation Wash Buffer; incubation at 57°C for 5 minutes; buffer removal. Finally, we pipetted 150µl of 2X SSC Buffer.

Tissue removal and Probe release - Visium CytAssist

The Visium CytAssist is a compact, bench top instrument that automates the transfer of transcriptomic analytes from standard microscope slides to Visium ones, enabling spatial profiling insights from more samples. The CytAssist Spatial Gene Expression Slide 6.5 mm has 2 Capture Areas. Each Capture Area is 6.5 x 6.5 mm and defined by a fiducial frame (fiducial frame + Capture Area is 8 x 8 mm). The Capture Area has ~5,000 barcoded spots. Each spot has the following oligos: Illumina TruSeq partial read 1 sequencing primer, 16 nucleotides (nt) Spatial Barcode, 12 nt unique molecular identifier (UMI), 30 nt poly(dT) sequence (captures ligation product). Each Capture Area on the CytAssist Spatial Gene Expression Slide is surrounded by a spacer. This spacer creates a reaction chamber that facilitates proper reagent addition and creates a seal between the CytAssist Spatial Gene Expression Slide and the Tissue Slide. The active surface of the slide is defined by a readable label that includes the serial number. We removed the slides from the Tissue Slide Cassette and stained the tissues again with H&E to ensure that the region of interest fit within the Capture Area of a Visium CytAssist Slide (6.5mm X 6.5mm) by overlaying the microscope and Visium slides. We placed on the top part of the CytAssist instrument the slides with the stained tissues and on the bottom part the Visium CytAssist Slide. On the latter, we added 4.7µl of Tissue Removal Enzyme and 70.3µl of Probe Release Mix, closed the lid of the instrument, and started the run at 37°C for 30 minutes.

Probe extension and elution

After the probe release and binding on Visium CytAssist Slide, we rinsed each Capture Area with 1 ml 2X SSC buffer for 3 times to wash, placed the Visium CytAssist Slide in the Visium Cassette, added 75µl of Probe Extension mix and placed on the Low-Profile Thermocycler Adapter on the pre-heated thermal cycler at 45°C for 15 minutes. Subsequently, we aliquoted 100µl of 2X SSC Buffer, removed it, and pipetted 50µl of 0.8M of KOH for 10 minutes at room temperature. Finally, we transferred all solutions for each sample containing the ligation product to a tube with 3µl of Tris-HCl pH 8.0.

Pre-amplification and solid phase reversible immobilization technology (SPRIselect)

The pre-amplification step is useful to generate ample material for library construction and amplification of barcoded ligation products. We added 47µl of Pre-Amplification Mix containing the universal TS primers B mix and incubated in a thermal cycler with the following protocol: 98°C for 3 minutes (step 1), 98°C for 15 seconds (step 2), 63°C for 20 seconds (step 3), 72°C for 30 seconds (step 4) and 72°C for 1 minute (step 5). Go from Step 2 to 4, repeat 9 times for a total of 10 cycles. Then, we pipetted 120 µl of SPRIselect reagent, incubated 5 min at room temperature, placed on the magnet High until the solution clears, removed the supernatant, putted 300µl of 80% ethanol to the pellet. After 30 seconds, we removed it and aliquoted 200µl of 80% ethanol to the pellet. We waited 30 seconds, removed the ethanol, and placed it on the magnet Low. Finally, we loaded 150µl of Buffer EB, incubated 2 minutes at room temperature, placed the tubes on the magnet High and transferred the total volume to new tubes.

Library preparation

The pre-amplification step determines the cycle number of each sample, and the appropriate sample index sets to ensure that no sample indices overlap in a multiplexed sequencing run in the following GEX sample index PCR. In each well of a qPCR plate, we putted 9µl of qPCR Mix and 1µl of 1:5 diluted samples and placed the plate in the thermal cycler with the following program: 98°C for 3 minutes (step 1), 98°C for 5 seconds (step 2), and 63°C for 30 seconds (step 3). Go to step 2 for 29 times (total of 30 cycles) and record the Cq Value for each sample. Subsequently, we added 70µl of Amplification Master Mix to 25µl of pre-amplificated samples and 5µl of individual Dual Index TS Set A containing a mix of one unique i7 (Index 1) and one unique i5 (Index 2) sample index that was recognized for sequencing. We incubated in a thermal cycler with the following protocol: 98°C for 3 minutes (step 1), 98°C for 15 seconds (step 2), 63°C for 20 seconds (step 3), 72°C for 30 seconds (step 4) and 72°C for 1 minute (step 5). Go from step 2 to 4 and use the Cq Value previously defined + 2 as the total number of cycles. Then, we proceeded with the GEX Post-Sample Index PCR Cleanup – SPRIselect by loading 85µl of SPRIselect Reagent and incubating 5 min at room temperature. After placing on the magnet High until the solution clears, we removed the supernatant and aliquoted 200µl 80% of ethanol to the pellet. After 30 seconds, we removed the ethanol and repeated the wash. We placed on the magnet Low, added 27µl of Buffer EB, incubated 2 minutes at room temperature, placed on the magnet Low until the solution cleared, and transferred 25µl sample to a new tube. We diluted the samples 1:50 run 1µl of sample on an Agilent Bioanalyzer High Sensitivity chip. If the peak is too small or flat, retry with a lower dilution. The expected average fragment size is 240 bp.

RNA isolation and RNA quality assessment

RNA Integrity Number (RIN) was calculated on the collected sections before placing them on the Visium Spatial slide, and a value ≥ 7 was considered adequate. We cryosectioned tissue sections from the OCT-embedded tissue block, each at 10μm thickness, and placed them in a microcentrifuge tube. RNA was isolated according to the RNeasy® Mini Kit Qiagen Handbook (HB-0435-006), and RIN was calculated using either Agilent RNA 6000 Nano.

Sequencing

Visium Spatial Gene Expression - libraries comprise standard Illumina paired-end constructs which begin with P5 and end with P7. 16 bp Spatial Barcodes are encoded at the start of TruSeq Read 1, while i7 and i5 sample index sequences are incorporated as the index read. TruSeq Read 1 and Small RNA Read 2 (Read 2S) are standard Illumina sequencing primer sites used in paired-end sequencing. TruSeq Read 1 are used to sequence the 16 bp Spatial Barcode and 12 bp UMI. Small RNA Read 2 (Read 2S) is used to sequence the Ligated Probe Insert. Sequencing these libraries produces a standard Illumina BCL data output folder. Calculating sequencing depth requires estimating the approximate Capture Area (%) covered by tissue. This may be performed by using the Visium Manual Alignment Wizard in Loupe Browser for a more accurate measurement. The number of spots covered by tissue was displayed during the "Identify Tissue" step and the total number of spots/Capture Area should be adjusted based on the Capture Area size (the number of spots for 6.5 mm slides is 4,992).

Visium Data Processing

In this study, sequencing reads were aligned and quantified using the *spaceranger* count pipeline (10x Genomics v2.1.1), mapping to the human reference genome (GRCh38). Tissue-covered areas on the slides were manually annotated to define the spatial capture regions. All downstream analyses were performed using the Seurat package (v5.0.0), with default parameters unless otherwise specified . The Seurat package was used to analyze and cluster the spots. The VlnPlot function was used to inspect the distribution of UMI counts across spatial spots. For each sample, data normalization and scaling were performed using the SCTransform function, regressing out mitochondrial (mt) gene content and spatial read depth. Dimensionality reduction was conducted using RunPCA, followed by neighborhood graph construction (FindNeighbors, dims = 1:20), clustering (FindClusters, res=0.8), and visualization via RunUMAP (dims = 1:20).

After normalization, samples were merged and integrated using Seurat's IntegrateLayers function, based on Canonical Correlation Analysis (CCA). The integrated dataset was again processed with FindNeighbors (dims = 1:30) and FindClusters (res=0.5) across multiple resolution values to identify

consistent and biologically meaningful spatial domains. Visualization was performed using RunUMAP (dims = 1:30). The top high variable genes were extracted from principal component analysis (PCA) to reduce the dimensionality of the data, and the top 15 significant principal components were subjected to cluster analysis at 0.5 resolution. The spatial future plot visualizes clusters from 0 to 7 and marker genes for cell clustering deconvolution were identified from a marker gene list filtered using $p<0.05$ and log2 fold change >0.5, as the threshold.

To assess the activity of predefined gene sets (signatures), module scores were calculated using Seurat's AddModuleScore function, with 100 control genes sampled per module to account for technical variability. Gene expression patterns were visualized using normalized counts with the pheatmap R package (v1.0.12) (Kolde R (2025). pheatmap: Pretty Heatmaps. R package version 1.0.13, https://github.com/raivokolde/pheatmap). Heatmaps of marker genes were generated using Seurat's DoHeatmap function. Spatial distributions of clusters, individual genes, and gene signatures were visualized using Seurat's SpatialFeaturePlot, SpatialDimPlot, RidgePlot, and DotPlot functions.

Enrichment analysis results were conducted with the ggplot2 package (v3.4.4) (Create Elegant Data Visualisations Using the Grammar of Graphics. https://ggplot2.tidyverse.org/index.html), and barplots of clusters, distributions for each sample were generated using the dittoSeq package (v1.13.3). Gene expression changes among clusters were tested setting "min.pct" to 0.1 and "logfc.threshold" to 0.5 to identify periportal and pericentral hepatocytes alongside cholangiocytes, endothelial cells, hepatic stellate cells and immune cells. Cell type identity was identified by Gene Ontology (GO) enrichment analysis, while pathways were collected from the KEGG database. Only pathways with a number of genes between 10 and 250 were considered. Over representation analysis was performed using the hypergeometric test and adjusted p-values (FDR) were obtained by the Benjamini–Hochberg procedure. To perform gene functional clustering, a "significant pathway similarity" graph was defined considering pathways enriched in DEGs (FDR < 0.05 and at least 3 DEGs) and the overlap coefficient as similarity measure between two pathways (based on their composition in DEGs).

Seahorse assay

We conducted the Seahorse Assay in frozen hepatic biopsies of n=14 patients (n=7 I148M, no; n=7 I148M, yes) belonging to the Validation cohort. The Seahorse procedure is available in previous study by Paolini E, et al. [10].

Transcriptomic analysis

Bulk RNA-seq analysis was performed in 45 hepatic samples (n=18 I148M, no; n=26 I148M, yes) of MASLD patients belonging to the Validation cohort. The experimental protocol of transcriptomic analysis is described in .

Portal risk score (PRS)

Since NAS score is unable to zone steatosis, necroinflammation and ballooning, we decided to build a novel scoring strategy that integrates NAS with portal disease, PNPLA3 GG and transaminases (LogAST). Specifically, in the Validation cohort (n=100) NAS was modeled as continuous value and weighted by the regression coefficient (beta1) of its association with periportal fibrosis in multivariable regression model adjusted for gender, age, BMI and T2D. LogAST was modeled as continuous value and weighted by the regression coefficient (beta3) of its association with periportal fibrosis in multivariable regression model adjusted as above. Conversely, PNPLA3 was modeled as a binary variable (GG vs GC+CC) and was weighted by the regression coefficient (beta2) of its association with periportal fibrosis in multivariable regression model adjusted for the same parameters.

The final score was calculated as follows:

Portal risk score=(beta1*NAS)+beta2*PNPLA3GG+beta3*logAST

where PNPLA3GG is=1 when patients are homozygous and =0 when patients are wild type or heterozygous, and beta1=0.48 and beta2=1.62, beta3=1.52.

The ability to discriminate fibrosis>2 was assessed by using receiver operating characteristic curve (ROC) analysis and compared with NAS, PNPLA3GG, logAST alone. Sensitivity and specificity were calculated at optimal cut-offs (Youden index, a statistic parameter that evaluates the discriminative performance of a diagnostic test). Finally, the score was tested in the large independent historical cohort of 1466 patients to assess robustness and reproducibility of the results (Independent retrospective cohort, [26].

Supplementary results

Spatial transcriptomic analysis identifies different hepatic populations in PNPLA3-WT and PNPLA3-I148M samples

Metabolic alterations within different zones could impair liver physiology, resulting in higher susceptibility to hepatic disorders [21]. Notably, the I148M variant led to altered lipid and glucidic signaling promoting steatosis onset, which in turn triggers mt-dysfunction and promotes advanced damage. Therefore, to deepen how the PNPLA3 variant impacts on metabolic zonation, we conducted an experiment of spatial transcriptomics in liver biopsies of WT and I148M homozygous patients featured by a similar disease severity. We exploited Loupe Browser v8.1.2 to zone HEPs and their metabolic signatures in specimen areas. The analyses were conducted after the manual selection of the spots in the hepatic tissues followed by the quality control based on UMI counts and number of detected genes (***Figure S1A-C***). By exploiting Loupe-marker gene list (***Table S2;*** avg_log2FC>0.5, p_val_adj<0.05), we outlined the signatures of cell-type populations and hepatic zonation. The spatial future plot revealed in both WT and I148M (***Figure S1D-E)*** hepatic biopsies the presence of HEPs as the main cell type alongside endothelial cells, hepatic stellate cells (HSCs), cholangiocytes, and immune cells.

Tables

Table S1: Clinical features of MASLD patients belonging to the Discovery (n=4) and Validation (n=100) cohorts

	Discovery cohort (n=4)	*Validation cohort (n=100)*
Sex, M	1(25)	51 (51.1)
Age, years	43±10.67	50.56±12.84
BMI, kg/m2	33.25±3.36	34.27±8.49
IFG/T2D, yes	0 (0.0)	28 (28.2)
HOMA-IR	6.25±1.55	4.64±3.43
Total cholesterol, nmol/L	5.66±0.10	5.15±1.09
LDL cholesterol, nmol/L	4.02±0.53	3.21±0.93
HDL cholesterol, nmol/L	1.36±0.29	1.33±0.36
Triglycerides, nmol/L	0.94±0.09	1.56±0.94
ALT, IU/L	25{14.85-35.14}	44.66{40.49-48.83}
AST, IU/L	39{10.86-67.10}	32.30{29.68-34.93}
NAS>4	1(25)	56 (21.0)
PNPLA3, GG yes	2(50)	34 (34)

Values are reported as mean±SD, number (%) or median {IQR}, as appropriate. BMI: body mass index; IFG: impaired fasting glucose; T2D: type 2 diabetes; HDL: high density lipoprotein; LDL: low density lipoprotein; ALT: alanine aminotransferase; AST: aspartate aminotransferase. Variables with skewed distribution were logarithmically transformed before analyses.

Table S2. Gene list for cell-type identification, zonation and metabolic signatures

**Cell types-signature**	**Genes**
**Hepatocytes**	*TTR, HNF4A, APOC3, ADH1B, FGB, HAMP*
**Hepatic stellate cells**	*RELN, LAMA, COLEC11*
**Cholangiocytes**	*HNF1b, PKHD1, TSPAN15*
**Endothelial cells**	*CLEC4G, SYNM, TINAGL1*
**Immune cells**	*SP110, TRIP4, OCEL1, OTC*
**Zonation-signature**	**Genes**
**Hepatocytes-Portal zone (PZ)**	*HAL, SDS*
**Hepatocytes-Central zone (CZ)**	*GLUL, CYP3A4*
**Metabolic-signature**	Genes
**Lipid metabolism**	*FASN, ACACA, DGAT1, DGAT2*
**Glucose metabolism**	*GPI, G6PD, ADH1A, PC*
**Mitochondrial activity**	*ALDH2, RMDN3, MTARC2*

List of genes (avg_log2FC>0.5, p_val_adj<0.05) for cell types-signature, zonation-signature and metabolic-signature spatially represented as LogNorm, that displays the UMI counts normalized to the total number of UMI counts *pe*r spot to compare the summed expression levels of all features in the list (Sum) between spots.

Table S3. Association between the I148M PNPLA3 variant (GG homozygous carriers) and panlobular steatosis, periportal inflammation and periportal fibrosis assessed histologically in MASLD patients (n = 100)

	Panlobular steatosis			Periportal inflammation			Periportal fibrosis		
	OR	95% CI	Pvalue	OR	95% CI	Pvalue	OR	95% CI	Pvalue
**Sex**	0.61	0.16-2.32	0.47	0,28	0.05-1.51	0.11	0.73	0.21-2.55	0.63
**Age**	1.02	0.96.1.08	0.45	1.01	0.95-1.05	0.87	0.43	0.93-1.03	0.55
**BMI**	0.99	0.91-1.07	0.82	1.09	0.99-1.19	0.05	0.98	0.91-1.06	0.67
**IGT/DIAB**	1.84	0.43-7.76	0.40	1.89	0.42-8.49	0.39	3.32	0.83-12.14	0.08
**NAS>4**	2.26	0.66-7.73	0.19	6.59	1.15-37.56	**0.01**	8.44	2.55-27.98	**0.0002**
PNPLA3, GG	16.23	4.12-63.89	**<.0001**	10.76	1.78-64.84	**0.0028**	4.33	1.23-15.15	**0.0182**

Values were obtained by nominal regression analysis (for panlobular steatosis, periportal inflammation and periportal fibrosis) adjusted for sex, age, BMI, IGT/T2D and NAS>4. Values of p<.05 are significant.

Table S4. Association between mitochondrial oxygen consumption rate (OCR) and periportal fibrosis in MASLD patients stratified according to I148M PNPLA3 variant (n =14)

**PNPLA3 I148M, yes (n=7)**	OCR		
	ß	95% CI	Pvalue
**Sex**	1713	-2142.05-5568.06	0.33
**Age**	-107.46	-411.78-196.86	0.43
Periportal fibrosis	-2565.26	-522.13--4608.37	**0.02**
**PNPLA3 I148M, no (n=7)**	**OCR**		
	ß	95% CI	Pvalue
**Sex**	3392.27	-3198.10-9982.65	0.26
**Age**	420.75	20.00-821.49	**0.042**
Periportal fibrosis	3590.39	7002.89-177.88	**0.041**

Values were obtained by nominal regression analysis for periportal fibrosis adjusted for sex and age in PNPLA3 I148M carriers (n=7) and non-carriers (n=7) . Values of p<.05 are significant.

Table S5. Association between PNPLA3 expression and periportal fibrosis in MASLD patients stratified according to I148M PNPLA3 variant (n = 45)

**PNPLA3 I148M, yes (n=26)**	**PNPLA3 expression**		
	ß	95% CI	Pvalue
**Sex**	-0.49	-1.13-0.14	0.12
**Age**	0.03	-0.005-0.07	0.09
Periportal fibrosis	0.59	0.20-0.98	**0.004**
**PNPLA3 I148M, no (n=18)**	**PNPLA3 expression**		
	ß	95% CI	Pvalue
**Sex**	-0.41	-1.28-0.45	0.32
**Age**	0.04	-0.06-0.15	0.41
Periportal fibrosis	0.45	0.70-1.16	0.42

Values were obtained by nominal regression analysis for periportal fibrosis adjusted for sex and age in PNPLA3 I148M carriers (n=26) and non-carriers (n=18) . Values of p<.05 are significant.

Table S6. Receiver operating characteristic (ROC) analysis of PRS and NAS, PNPLA3GG, LogAST alone to predict advanced fibrosis in the Validation cohort (n=100)

**VALIDATION COHORT (n=100) - Fibrosis>2 - NAS<4**				
	AUC	Sensitivity	1-Specificity	Youden index
NAS	0.80	77	23	0.54
PNPLA3GG	0.75	69	19	0.50
LogAST	0.77	85	30	0.54
Beta1*NAS+beta3*logAST	0.84	85	27	0.57
Beta1*NAS+beta2*PNPLA3GG	0.86	92	36	0.56
Portal Risk Score (PRS)	0.89	93	20	0.73

The table includes the area under the curve (AUC), sensitivity, 1-specificity and Youden index parameters, which is statistic parameter that evaluates the discriminative performance of a diagnostic test. P=0.11 PRS vs NAS; P=0.0093 PRS vs PNPLA3GG; P=0.03 PRS vs logAST.

Table S7. Receiver operating characteristic (ROC) analysis of PRS and NAS, PNPLA3GG, LogAST alone to predict advanced fibrosis in the Independent retrospective cohort (n=1466)

INDEPENDENT RETROSPECTIVE COHORT (n=1466) - Fibrosis>2 - NAS<4				
	AUC	Sensitivity	1-Specificity	Youden index
NAS	0.77	71	25	0.46
PNPLA3GG	0.65	41	11	0.30
LogAST	0.76	80	28	0.51
Beta1*NAS+beta3*logAST	0.82	77	23	0.54
Beta1*NAS+beta2*PNPLA3GG	0.81	83	32	0.50
Portal Risk Score (PRS)	0.85	87	23	0.60

The table includes the area under the curve (AUC), sensitivity, 1-specificity and Youden index parameters, which is statistic parameter that evaluates the discriminative performance of a diagnostic test. P=0.0089 PRS vs NAS; P<0.0001 PRS vs PNPLA3GG; P=0.0048 PRS vs logAST.

Supplementary Figures

Figure S1

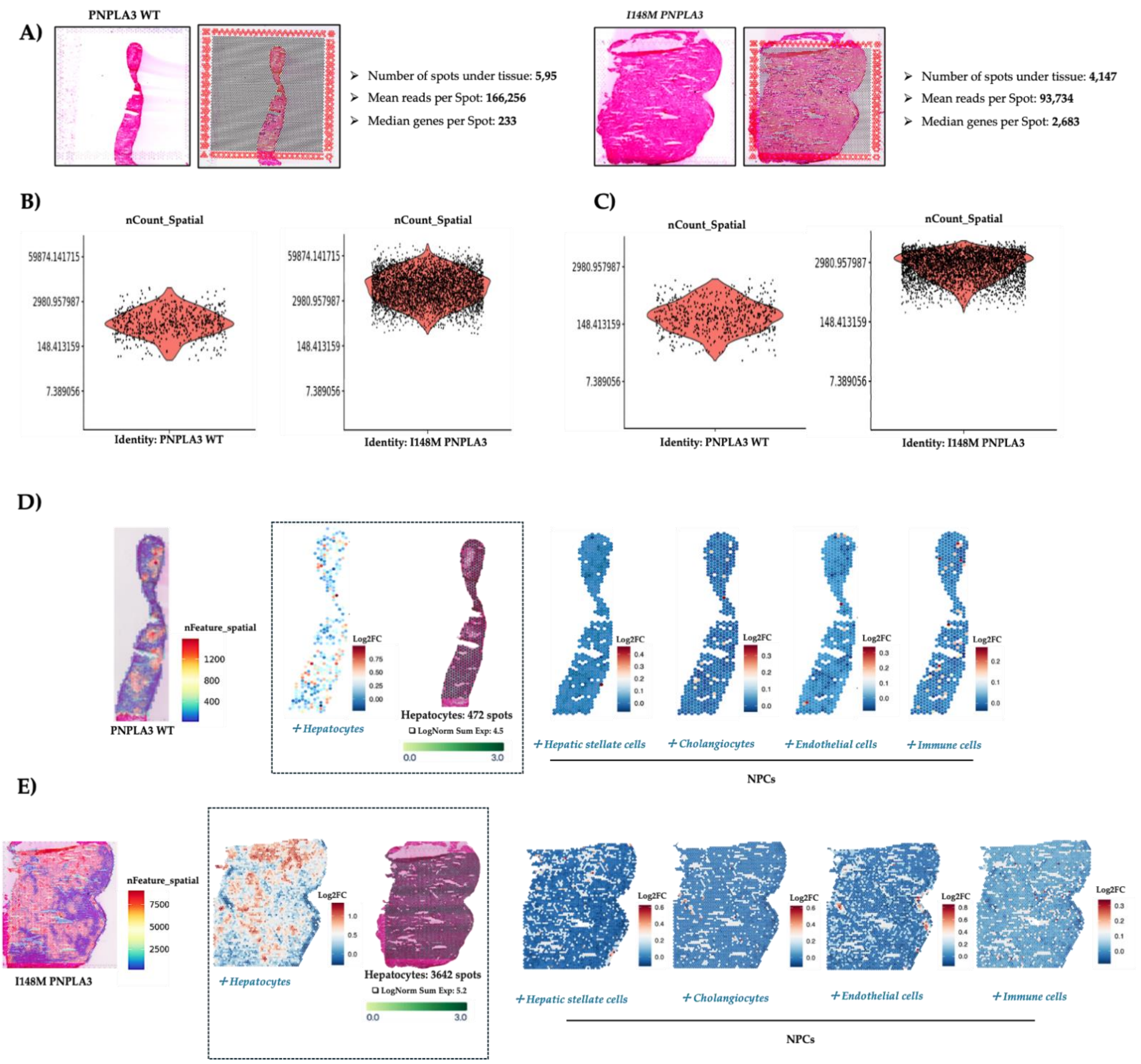

Figure S1. LOUPE analysis: **A)** Manual selection of the spots in the hepatic tissues by using Loupe browser (v.8.1.2); **B-C)** Violin plot of the distribution of Unique Molecular identifier (UMI) counts and genes number for WT and I148M PNPLA3 samples; ***D-E)*** The spatial future plot revealed in both WT and I148M PNPLA3 hepatic biopsies the presence of HEPs as the main cell type alongside endothelial cells, hepatic stellate cells (HSCs), cholangiocytes, and immune cells (LOUPE): avg_log2FC>0.5, p_val_adj<0.05.

Figure S2

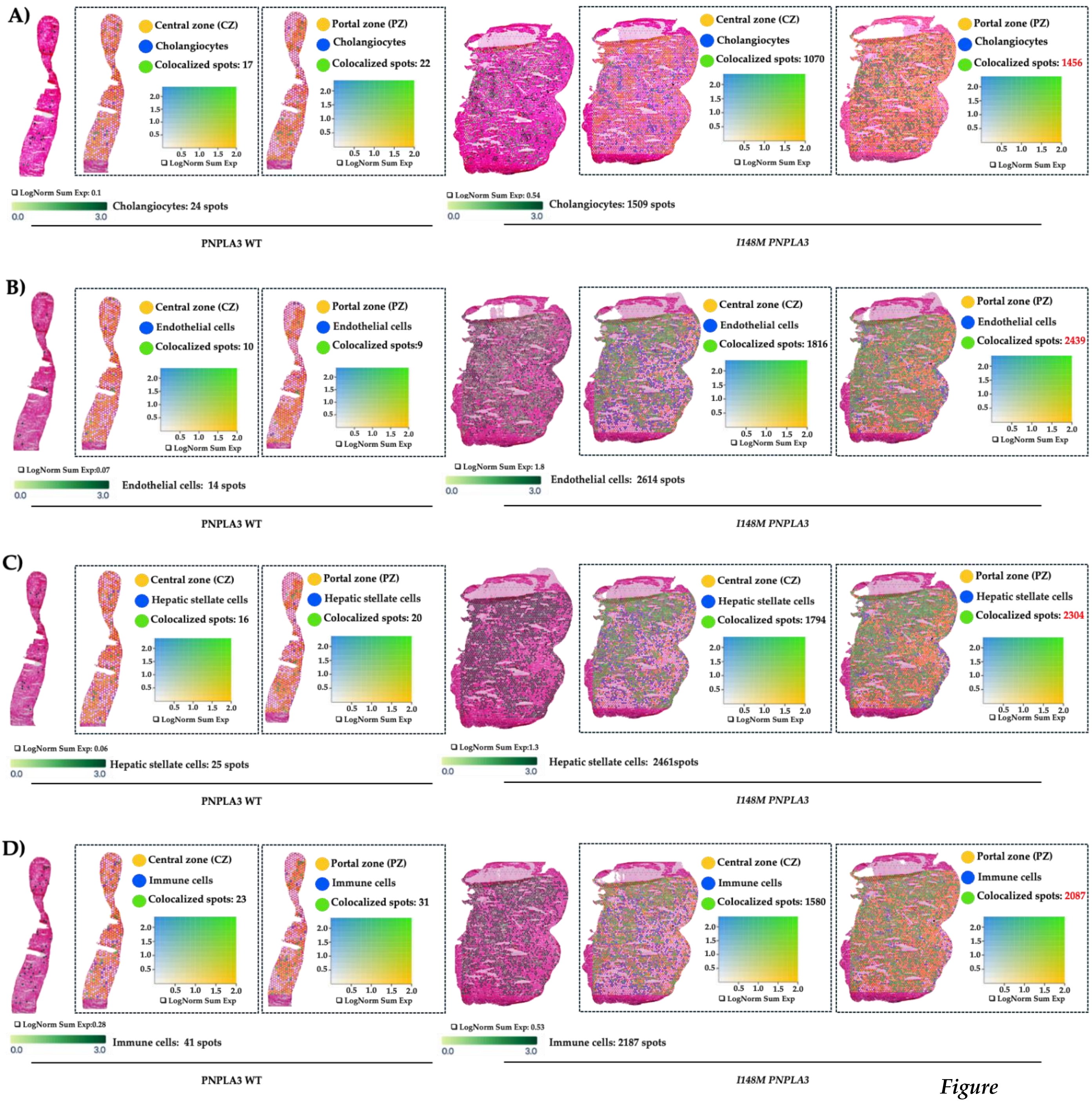

Figure S2. LOUPE analysis colocalized non-parenchymal cells with periportal hepatocytes of I148M samples: **A)** Spatial feature plots show the expression of classical genes of cholangiocytes (CHOLs) (left panel) across the liver sections of WT and I148M PNPLA3 (left panel). The expression intensity is represented by the color scale. CHOLs were zoned by co-expressing UMI counts of Portal zone (PZ) or Central zone (CZ) signatures (**Table S2;** squared right panel) as LogNorm; **B)** Spatial feature plots show the

expression of classical genes of endothelial cells (ENDOs) (left panel) across the liver sections of WT and I148M PNPLA3 (left panel). The expression intensity is represented by the color scale. ENDOs were zoned by co-expressing UMI counts of PZ or CZ signatures (**Table S2;** squared right panel) as LogNorm; **C)** Spatial feature plots show the expression of classical genes of hepatic stellate cells (HEPs) (left panel) across the liver sections of WT and I148M PNPLA3 (left panel). The expression intensity is represented by the color scale. HSCs were zoned by co-expressing UMI counts of PZ or CZ signatures (**Table S2;** squared right panel) as LogNorm; **D)** Spatial feature plots show the expression of classical genes of immune cells (left panel) across the liver sections of WT and I148M PNPLA3 (left panel). The expression intensity is represented by the color scale. Immune cells were zoned by co-expressing UMI counts of PZ or CZ signatures (**Table S2;** squared right panel) as LogNorm. LogNorm displays the UMI counts normalized to the total number of UMI counts *pe*r spot to compare the summed expression levels of all features in the list (Sum) between spots.

The co-localization is evidenced by green spots.

Figure S3

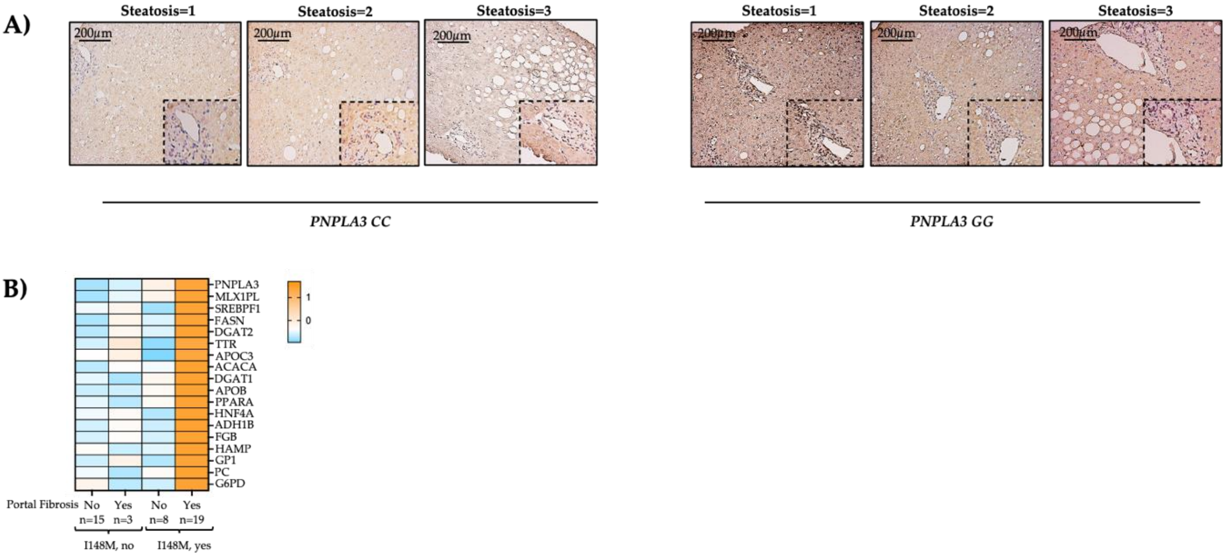

***Figure S3. PNPLA3 is highly expressed in I148M patients in periportal zone*: A)** PNPLA3 staining by IHC was performed in hepatic tissues of PNPLA3 CC and PNPLA3 GG subjects. Representative images of periportal areas are zoomed in dashed rectangles (Original magnification 200X, scale bar 200μm). **B)** Heatmap represent the average relative expression (z-score) of genes belonging to the lipid and glucose metabolism, in n=45 MASLD patients stratified according to the presence of portal fibrosis and PNPLA3 I148M variant. The orange shading represents the induction, whereas the blue shading indicates the repression. Z-scores were calculated from the values obtained by applying DESeq2 variance, stabilizing

transformation to gene counts. P-values were corrected by Benjamini–Hochberg false discovery rate and only genes with adjusted p values lower than 0.05 are represented in the figure.

References

10. Paolini, E.; Longo, M.; Meroni, M.; Podini, P.; Maggioni, M.; Quattrini, A. A Defective Circulating Mitochondrial Bioenergetics Profile Reflects the Hepatic One and Outlines Genetic MASLD. *Antioxidants* **2025**, *14*, 618.

20. Kleiner, D.E.; Brunt, E.M.; Van Natta, M.; Behling, C.; Contos, M.J.; Cummings, O.W.; Ferrell, L.D.; Liu, Y.-C.; Torbenson, M.S.; Unalp-Arida, A.; et al. Design and validation of a histological scoring system for nonalcoholic fatty liver disease. *Hepatology* **2005**, *41*, 1313–1321.

21. Soto-Gutierrez, A.; Gough, A.; Vernetti, L.A.; Taylor, D.L.; Monga, S.P. Pre-clinical and clinical investigations of metabolic zonation in liver diseases: The potential of microphysiology systems. *Exp. Biol. Med.* **2017**, *242*, 1605–1616.

26. Longo, M.; Paolini, E.; Meroni, M.; Ripolone, M.; Napoli, L.; Gentile, F.; Cespiati, A.; Trombetta, E.; Lombardi, R.; Maggioni, M.; et al. Artificial intelligence as a ploy to delve into the intricate link between genetics and mitochondria in patients with MASLD. *JHEP Rep.* **2025**, *7*, 101539.

34. Meroni, M.; Dongiovanni, P. PNPLA3 rs738409 Genetic Variant Inversely Correlates with Platelet Count, Thereby Affecting the Performance of Noninvasive Scores of Hepatic Fibrosis. *Int J Mol Sci* **2023**,*24:15046. doi: 10.3390/ijms242015046.*
